# Protective Effects of Beta-3 Adrenoceptor Agonism on Mucosal Integrity in Hyperoxia-Induced Ileal Alterations

**DOI:** 10.3390/antiox13070863

**Published:** 2024-07-18

**Authors:** Patrizia Nardini, Virginia Zizi, Marta Molino, Camilla Fazi, Maura Calvani, Francesco Carrozzo, Giorgia Giuseppetti, Laura Calosi, Daniele Guasti, Denise Biagini, Fabio Di Francesco, Luca Filippi, Alessandro Pini

**Affiliations:** 1 Department of Experimental and Clinical Medicine, University of Florence, 50139 Florence, Italy; patrizia.nardini@unifi.it (P.N.); virginia.zizi@unifi.it (V.Z.); marta.molino@unifi.it (M.M.); giorgia.giuseppetti@unifi.it (G.G.); laura.calosi@unifi.it (L.C.); daniele.guasti@unifi.it (D.G.); 2 Imaging Platform, Department Experimental and Clinical Medicine & Joint Laboratory with Department Biology, University of Florence, 50139 Florence, Italy; 3 Department of Pediatric, Meyer Children’s University Hospital, 50139 Florence, Italy; camilla.fazi@unifi.it; 4 Azienda Ospedaliera Universitaria Meyer, Istituto di Ricovero e Cura a Carattere Scientifico (IRCCS), 50139 Florence, Italy; maura.calvani@meyer.it; 5 Department of Health Science, University of Florence, 50139 Florence, Italy; francesco.carrozzo@unifi.it; 6 Department of Chemistry and Industrial Chemistry, University of Pisa, 56124 Pisa, Italy; denise.biagini@dcci.unipi.it (D.B.); fabio.difrancesco@unipi.it (F.D.F.); 7 Neonatology and Neonatal Intensive Care Unit, Department of Clinical and Experimental Medicine, University of Pisa, 56124 Pisa, Italy

**Keywords:** beta-3 adrenoceptor, beta-3 adrenoceptor agonism, BRL37344, hyperoxia, ileum, oxidative stress, oxygen, development, mucosa

## Abstract

Organogenesis occurs in the uterus under low oxygen levels (4%). Preterm birth exposes immature newborns to a hyperoxic environment, which can induce a massive production of reactive oxygen species and potentially affect organ development, leading to diseases such as necrotizing enterocolitis. The β3-adrenoreceptor (β3-AR) has an oxygen-dependent regulatory mechanism, and its activation exerts an antioxidant effect. To test the hypothesis that β3-AR could protect postnatal ileal development from the negative impact of high oxygen levels, Sprague–Dawley rat pups were raised under normoxia (21%) or hyperoxia (85%) for the first 2 weeks after birth and treated or not with BRL37344, a selective β3-AR agonist, at 1, 3, or 6 mg/kg. Hyperoxia alters ileal mucosal morphology, leading to increased cell lipid oxidation byproducts, reduced presence of β3-AR-positive resident cells, decreased junctional protein expression, disrupted brush border, mucin over-production, and impaired vascularization. Treatment with 3 mg/kg of BRL37344 prevented these alterations, although not completely, while the lower 1 mg/kg dose was ineffective, and the higher 6 mg/kg dose was toxic. Our findings indicate the potential of β3-AR agonism as a new therapeutic approach to counteract the hyperoxia-induced ileal alterations and, more generally, the disorders of prematurity related to supra-physiologic oxygen exposure.

## 1. Introduction

Mammalian organ development takes place in the uterus [1] at low oxygen tension (4%) and for many organs, including lungs, eyes, and gut, is completed postnatally at ambient oxygen levels (21%) [2,3,4,5]. Preterm birth exposes immature newborns to a relatively hyperoxic environment compared to the intrauterine one. This could alter the postnatal development of their organs, leading to diseases such as retinopathy of prematurity (ROP) [6], bronchopulmonary dysplasia (BPD) [7,8], and necrotizing enterocolitis (NEC) [4]. Although the long-term effects induced by hyperoxia may be relevant to infant life, little is known about the role of high oxygen levels in organ development, especially for the organs that continue to grow postnatally, such as the intestine [7]. Minimal research has shown that exposure to neonatal hyperoxia alters the development of the colon [9] by increasing its wall thickness and mucin production [7,9] and damages the distal small intestine by disrupting the ileal mucosal barrier [4,7], altering villous structures [10,11], and altering vascularization [12]. An important contributing factor in hyperoxia-induced damage is the massive production of reactive oxygen species (ROS), which compromises the intestinal epithelium’s integrity [4,11].

Considering the protective effect against oxidative stress induced by β3-adrenoreceptor (β3-AR) activation [13,14] and the relationship between its expression and oxygen levels [5], it is conceivable that β3-AR could be involved in the ileal alterations induced by hyperoxia. β3-AR belongs to a family of G-protein-coupled receptors, composed of α1-ARs, α2-ARs, and β1-ARs, β2-ARs and β3-Ars [15,16].

First discovered in adipose tissue in 1989 [17], β3-AR differs from the other subtypes concerning its expression profile and regulatory mechanism, being sensitive to oxygen levels. It is highly expressed in the developing embryo and fetus during hypoxic intra-uterine life [18,19,20,21]; conversely, β3-AR reduces its expression after birth [5,21,22] when newborns are exposed to ambient oxygen levels and persists in restricted human adult districts, including the adipose tissue [23], myocardium [24], pregnant myometrium [25], bladder [26], brain [27], retina, blood vessels, gallbladder [28], and gut [9,29]. It has also been demonstrated that exposure of the immature colon to hyperoxia alters its development process by reducing β3-AR-expressing cells [9]. In addition to its peculiar oxygen-dependent regulatory mechanism, β3-AR has been recently demonstrated to reduce oxidative stress by inhibiting NADPH oxidases [30], thus protecting the brain [31] and myocardium [13,30] from the detrimental effects of reactive oxygen species (ROS).

To substantiate the hypothesis that β3-AR could have a relevant role in protecting postnatal ileal development from the negative impact of high oxygen levels, we assessed the effects of BRL37344, a commonly used selective β3-AR agonist, in a neonatal rat model of hyperoxia-induced ileal damage.

## 2. Materials and Methods

### 2.1. Exposure to Hyperoxia and Normoxia and Drug Administration

This study was approved by the Ethical Committee of Florence University and the Italian Ministry of Health (Authorization N. 453/2021).

Pregnant Sprague–Dawley rats (Crl:CD(SD)) were individually housed in cages under consistent environmental and nutritional conditions at 25 ± 2 °C. They were exposed to alternating 12 h light and dark cycles and had unrestricted access to food and water. After delivery, the litters were immediately pooled and randomly assigned to either oxygen-enriched atmosphere (hyperoxia, 85%) treatments or ambient oxygen levels (normoxia, 21%). Hyperoxia was maintained in an animal chamber (BioSpherix, Parish, NY, USA), where oxygen levels were monitored using a ProOx P110 probe (BioSpherix). Some experimental groups were treated with the selective β3-AR agonist BRL37344 (Merck, Darmstadt, Germany), administered subcutaneously every 12 h from birth (P0) to day 14 (P14). The lactating dams were swapped between normoxia and hyperoxia every 24 h to avoid oxygen toxicity. The pups were randomly assigned to six groups: (1) normoxia controls (the pups were reared to 21% oxygen and untreated); (2) normoxia + BRL37344 (the pups were reared in 21% oxygen and administered with 3 mg/kg BRL37344); (3) hyperoxia controls (the pups were exposed to 85% oxygen and untreated); (4) hyperoxia + BRL37344 (1 mg/kg) (the pups were reared in 85% oxygen and treated with 1 mg/kg BRL37344); (5) hyperoxia + BRL37344 (3 mg/kg) (the pups were exposed to 85% oxygen and treated with 3 mg/kg BRL37344); and (6) hyperoxia + BRL37344 (6 mg/kg) (the pups were reared in 85% oxygen and administered with 6 mg/kg BRL37344). Body weight was measured daily before the injections. At the end of the experiment, the animals were sacrificed by sodium pentobarbital (800 mg/kg i.p.), and terminal ileum samples were taken 3 cm from the caecum. Specimens were then washed in 0.1 M phosphate-buffered saline (PBS) at pH 7.4 and processed for biochemical and morphologic evaluations.

### 2.2. Sample Collection and 5-Epi-5-F_2t_-IsoP Measurement

Whole blood was collected in EDTA tubes and centrifuged at 1500× *g* for 10 min at 25 °C to separate plasma, which was stored in aliquots at −80 °C until analysis. Before storage, the antioxidant butylhydroxytoluene (BHT, 15 mg/mL in methanol, 1:100 sample volume ratio) was added to prevent lipid peroxidation. The free content of the plasma level of isoprostane 5-*epi*-5-F_2t_-IsoP, a reliable marker of oxidative stress [32], was measured with a Micro-Extraction by Packed Sorbent (MEPS) liquid chromatography–tandem mass spectrometry platform (MEPS-LC-MS/MS) as previously reported [33].

### 2.3. Flow Cytometric Analysis

Cell suspensions were extracted from frozen ileum samples using the Multi Tissue Dissociation Kit 2 (Miltenyi Biotec, Bergisch Gladbach, Cologne, Germany) and subjected to flow cytometry analysis. Cells were immunolabeled with the FITC-conjugated anti-CD45 antibodies [OX-1] FITC (GTX43587, GeneTex, Irvine, CA, USA) and anti-β3-AR A594 antibodies (Bioss, Woburn, MA, USA), diluted 1:50. The labeled cells were analyzed and quantitated using a MACSQuant Analyzer 10 Flow Cytometer (Miltenyi Biotec) and Flowlogic 8.7 software (Miltenyi Biotec).

### 2.4. Histological Evaluations

Ileal specimens were excised as previously reported and divided into three segments, each 1 cm long. The middle and terminal ones were used for the whole mounts and paraffin-embedded cross-sections, respectively. For whole-mount preparation, the tissue was transferred to a silicone-coated plate containing ice-cold PBS and longitudinally opened using spring scissors. The sample was then pinned down and gently stretched using insect pins onto the silicone-coated plate so that the villi were facing upwards and the tissue was as flat and straight as possible [34]. The samples were fixed at +4 °C using 4% paraformaldehyde (PAF) for 2 h and then stored in PBS with 0.1% sodium azide until the immunofluorescence analysis.

The samples selected for paraffin-embedded cross-section were fixed at 4 °C overnight in 4% PAF, dehydrated in graded ethanol, cleared in Bio-clear (Bio-Optica, Milan, Italy), and embedded in paraffin. Then, 5 µm thick sections were stained with hematoxylin and eosin (H/E) to evaluate the mucosal morphology and with PAS reaction to quantify the mucin content. To minimize artefactual staining differences, all sections were stained in a single session. Examinations were performed by two independent observers (A.P., P.N.), blinded to the experimental groups, on digital images acquired with a light microscope equipped with 20× and 40× objectives and a high-resolution camera (Leica DFC310 F× 1.4-megapixel, Leica Microsystems, Mannheim, Germany). The morphometric analysis of mucin content was performed on ten regions of interest (ROIs) randomly taken for each section, evaluating the PAS-positive areas using ImageJ with Java8 software (NIH, Bethesda, ML, USA).

### 2.5. Immunofluorescence Analysis

The immunofluorescence analysis was carried out on the whole-mount preparations and the cross-sections to evaluate the vascularization and the epithelial junctions, respectively.

The whole-mount preparations were permeabilized using a PBS-triton X solution (0.2% *v*/*v*) followed by incubation in a solution of BSA (3%, *m*/*v*)-PBS-triton X (0.1% *v*/*v*) for blocking the non-specific staining. Primary goat anti-rat CD31 (R&D; Minneapolis, MN, USA) was diluted 1:100 in PBS 1.5% BSA and incubated overnight at +4 °C under gentle shaking. Samples were rinsed with PBS and incubated in rotation with secondary anti goat-Alexa Fluor 594 (Jackson ImmunoResearch, Ely, UK) diluted 1:175 in PBS 1.5% BSA for 2 h at room temperature. After the final washing step with PBS, the strips were transferred into a droplet of the anti-fade DAPI mounting medium (Fluoroshield^TM^ with DAPI, Thermo Fisher Scientific, Waltham, MA, USA) dripped onto the microscope slide and oriented on their side to visualize the villus axis [34].

The visualization, acquisition, and quantification of the CD31 immunolabelled area were performed using, respectively, a Leica Stellaris 5 confocal microscope equipped with the 20× objective lens (Leica Microsystem) and Fiji with Java8 software (NIH). Confocal images were obtained at high resolution (1024 pixels × 1024 pixels × 10 µm), combining the mosaic mode to analyze the entire sample surface and z-stack mode to measure a fixed thickness of 10 µm per strip. The resulting mosaic z-stack images were acquired with a fixed laser intensity and z-stack range of 1 µm.

The segmentation process was performed on z-stack projection images as the result of the sum of all optical slices acquired per sample. Finally, the immunolabelling was quantified by thresholding the CD31 immunolabelled areas as regions of interest (ROIs) selected by the handmade selection. Results were expressed as the ratio of the CD31-positive and total analyzed areas [35].

The paraffin-embedded sections were rehydrated and treated for antigen retrieval using sodium citrate buffer (10 mM, pH 6) for 20 min at 90 °C. The sections were then rinsed in PBS, blocked with 3% bovine serum albumin (BSA, AppliChem, Darmstadt, Germany) in PBS to minimize non-specific binding, and incubated at 4 °C overnight with primary mouse anti-rat E-cadherin (Cell Signaling, Danvers, MA; USA) or primary rabbit anti-rat ZO-1 (Thermo Fisher Scientific, Waltham, MA, USA), both diluted 1:100 in PBS 1.5% BSA. After PBS washes, the samples were incubated with secondary anti-mouse-Alexa Fluor 594 or anti-rabbit-Alexa Fluor 488 (Jackson ImmunoResearch, West Grove, PA, USA) for 2 h at room temperature. The specimens were washed with PBS and mounted in DAPI anti-fade aqueous medium (Fluoroshield^TM^ with DAPI, Thermo Fisher Scientific). Negative control was performed by omitting the primary antibody. The immunolabeled sections were observed under the Leica Stellaris 5 confocal microscope equipped with a Leica Plan Apo 20× objective. The images were acquired using the parameters previously described for the whole-mount preparations. The mosaic z-stack images comprised merged tiles of the entire cross-section to cover the whole mucosal surface and stacks, which were captured every 1 µm to analyze the total sample thickness. The E-cadherin and ZO-1-positive areas were measured on the z-stack projection images using the threshold for selecting the appropriate immunolabelled area, and the results were expressed as the percentage of the E-cadherin- or ZO-1-positive area on the total mucosal analyzed area.

### 2.6. Transmission Electron Microscopy Analysis

Small fragments collected from the terminal ileum were fixed overnight in Karnovsky’s mixture at +4 °C. Samples were post-fixed in 1% OsO_4_ in phosphate buffer for 2 h at room temperature, dehydrated in graded acetone, passed in propylene oxide, and embedded in epoxy resin (Epon 812, Sigma-Aldrich, Darmstadt, Germany). Ultrathin sections (70 nm thick) were stained with UranyLess (Electron Microscopy Sciences, Hatfield, PA, USA) and alkaline bismuth subnitrate solutions and then examined under a JEM-1010 electron microscope (Jeol, Tokyo, Japan) at 80 kV. A MegaView III high-resolution digital camera equipped with AnalySIS 2.2 imaging software (Soft Imaging System, Muenster, Germany) was used to acquire the photomicrographs. Microvilli and cellular junctions were observed and acquired at 10,000× and 25,000× magnification for qualitative evaluations.

### 2.7. Statistical Analysis

Data are expressed as mean ± S.D. of at least 11 animals per group. Statistical analysis was carried out using a one-way analysis of variance (ANOVA) test followed by Tukey’s multiple comparison test. The normoxia + BRL37344 group was compared to the normoxia group (#), and the hyperoxia + BRL37344 groups were compared to both the hyperoxia (§) and normoxia groups (°), to evaluate the protection degree of BRL37344 from the adverse effects of hyperoxia. Statistical analysis was performed using GraphPad Prism 9.0 software (GraphPad, San Diego, CA, USA).

## 3. Results

### 3.1. Plasma Levels of 5-Epi-5-F_2t_-IsoP

**Figure 1:**
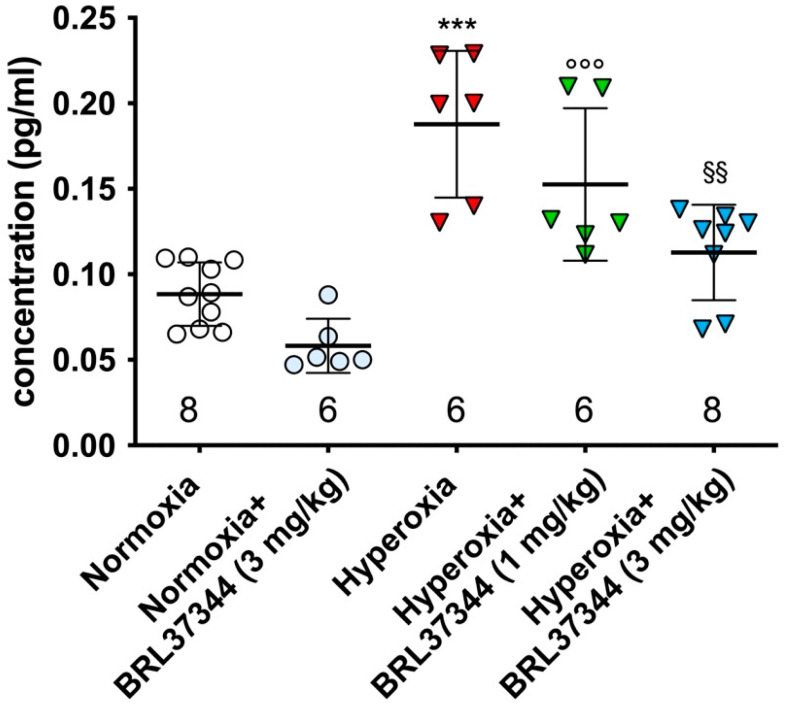
The plasma level of 5-*epi*-5-F_2t_-IsoP. The level of the isoprostane 5-*epi*-5-F_2t_-IsoP was significantly increased in the plasma of the hyperoxia-exposed control rats. Treatment with BRL37344 at 3 mg/kg restored the 5-*epi*-5-F_2t_-IsoP level to that of the normoxia control animals. Under normoxia, no differences were observed in the pups treated with BRL37344 or not. Values are expressed as mean ± S.D. *** *p* < 0.001 hyperoxia versus normoxia; °°° *p* < 0.001 hyperoxia + BRL37344 versus normoxia; §§ *p* < 0.01 hyperoxia + BRL37344 versus hyperoxia.

The peroxidation of arachidonic acid (AA) by free radicals leads to the production of F2-isoprostanes (F2-IsoPs). The 5-series F_2_-IsoPs are the most abundant [36] and are associated with cellular oxidant injury [36]. Exposure to neonatal hyperoxia significantly increased the plasma level of 5-*epi*-5-F_2t_-IsoP (Figure 1), an alteration that was completely prevented by 3 mg/kg of BRL37344. In contrast, the 1 mg/kg dose was ineffective (Figure 1). It must be pointed out that BRL37344 at the highest dose (6 mg/kg) caused the death of all the pups reared in hyperoxia within the first week, thus precluding any further analysis. No differences were found in normoxia-exposed pups, regardless of whether they were treated with BRL37344 or not (Figure 1).

### 3.2. β3-AR Expression on Ileal Resident Cells

**Figure 2:**
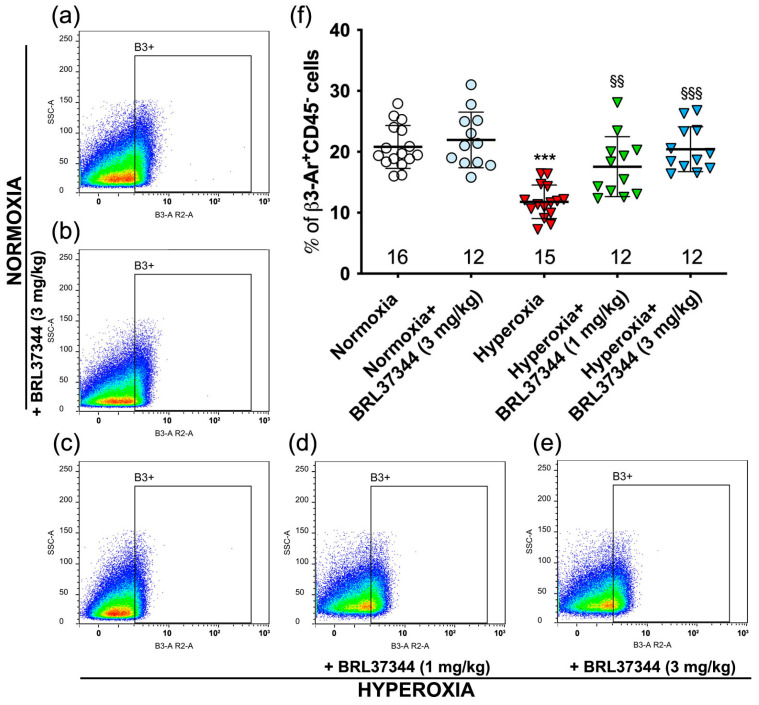
Effect of hyperoxia on β3-AR-positive ileal resident cells. (**a**–**e**) Flow cytometric plot of ileal cells expressing β3-AR (β3-AR^+^ CD45^−^ cells). (**f**) Statistical analysis of the cytofluorimetric plots. The number of β3-AR^+^ cells was significantly decreased under hyperoxia, and treatment with BRL37344 significantly averted this reduction. Under normoxia, no differences were shown in the pups treated with BRL37344. Values are expressed as mean ± S.D. *** *p* < 0.001 hyperoxia versus normoxia; §§ *p* < 0.01 and §§§ *p* < 0.001 hyperoxia + BRL37344 versus hyperoxia.

It is known that hyperoxia reduces the number of resident cells in the proximal colon that express β3-AR [9]. We wanted to find out whether hyperoxia had the same effect in the ileum. Flow cytometric analysis was carried out on cell suspensions isolated from the ileal specimens, and the pan-hematopoietic marker CD45 [37] was used to exclude inflammatory cells, which constitutively express β3-AR [38,39]. The analysis revealed a statistically significant reduction in the number of ileal β3-AR-positive cells (β3-AR^+^ CD45-cells) in the hyperoxia control mice compared with those reared in normoxia (Figure 2a,c,f). Administration of BRL37344 at both 1 and 3 mg/kg counteracted the hyperoxia-induced decrease in β3-AR-expressing cells (Figure 2a,c–f). Under normoxic conditions, BRL37344 did not alter the number of resident ileal cells that express β3-AR (β3-AR^+^ CD45-cells) (Figure 2a,b,f).

### 3.3. Body Weight

As previously reported, exposure to hyperoxia during early postnatal life causes significant body weight loss in rats [9]. After the experimental period (P14), the body weight of the pups raised in hyperoxia was notably lower than that of the normoxic ones. Administration of BRL37344 at both doses did not mitigate this outcome (Table 1).

### 3.4. Histological Assessment of Mucosal Morphology

**Figure 3:**
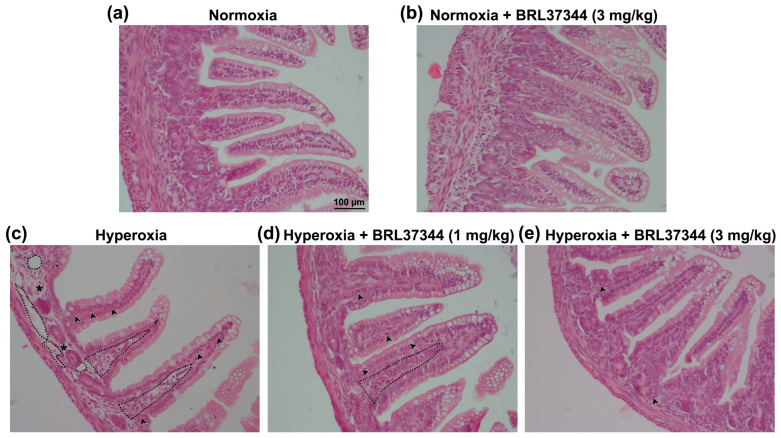
Histological evaluation of the effects of hyperoxia on ileal mucosal morphology. (**a**–**e**) Representative images of H/E-stained sections. (**b**) No differences were observed in the normoxia-exposed rats treated or not with BRL37344. (**c**) The hyperoxia-exposed controls displayed capillary and lacteal dilation (black dotted line) in the lamina propria, submucosal oedema (asterisk), and mixed leukocyte infiltration (arrowhead). (**d**) Rats exposed to hyperoxia and treated with 1 mg/kg BRL37344 also exhibited capillary dilation (black dotted line) and leukocyte infiltration (arrowhead). (**e**) Normal ileal morphology was preserved in the rats exposed to hyperoxia and treated with 3 mg/kg BRL37344, except for scattered infiltrating leukocytes (arrowhead). Scale bars = 100 µm.

The impact of exposure to hyperoxia on ileal mucosal morphology was qualitatively evaluated on H/E-stained sections. The pups reared in normoxia showed folded mucosal villi with continuous lining epithelium (Figure 3a). Instead, the hyperoxia-exposed controls exhibited capillary and lacteal dilation in the lamina propria, as well as signs of submucosal oedema and leukocyte infiltration, although the overall integrity of the surface epithelium seemed to be maintained (Figure 3c). Hyperoxia-exposed rats treated with 1 mg/kg of BRL37344 also displayed widened vessels and infiltrating leukocytes (Figure 3d), while only minor abnormalities, limited to a moderate inflammatory cell infiltration, were observed in hyperoxia-exposed rats treated with 3 mg/kg of BRL37344 (Figure 3e). Conversely, no differences in mucosal morphology were found in rats exposed to normoxia, whether or not they were treated with BRL37344 (Figure 3b).

**Figure 4:**
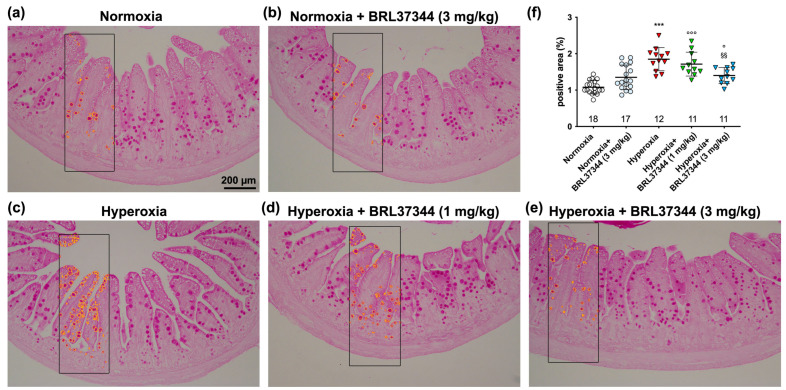
Quantitative analysis of PAS^+^ mucin-producing goblet cells. (**a**–**e**) Representative histologic images. (**f**) Morphometric analysis of PAS^+^ relative area. The exposure to hyperoxia significantly increased the percentage of PAS^+^ area (**a**,**c**,**f**) (yellow outlined in the inserts), an effect that was prevented by 3 mg/kg BRL37344 administration (**c**,**e**,**f**). No change was observed in the normoxia-reared pups treated or not with BRL37344 (**a**,**b**,**f**). Values are expressed as mean ± S.D. *** *p* < 0.001 hyperoxia versus normoxia; ° *p* < 0.05 and °°° *p* < 0.001 hyperoxia + BRL37344 versus normoxia; §§ *p* < 0.01 hyperoxia + BRL37344 versus hyperoxia. Scale bar = 200 µm.

The impact of high oxygen levels on mucin production was quantitatively evaluated by analyzing PAS-stained ileal sections [40]. A significant increase in the PAS^+^ relative area of goblet cells was observed in the hyperoxic controls (Figure 4a,c,f), indicating mucin over-production. Administration of 3 mg/kg of BRL37344 reduced significantly, although not completely, this hyperoxia-induced alteration, while the lowest 1 mg/kg dose was ineffective (Figure 4c–f). No differences were observed in the normoxic groups, suggesting that BRL37344 does not affect ileal mucin production per se (Figure 4a,b,f).

### 3.5. Immunofluorescent Analysis and Ultrastructural Evaluation of the Ileal Lining Epithelium

**Figure 5:**
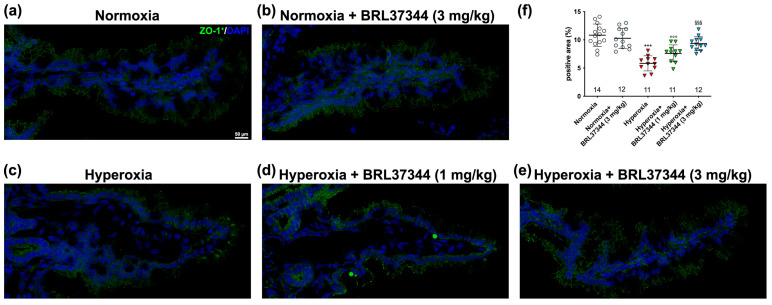
ZO-1 immunolabelling in the ileal mucosa. (**a**–**e**) Representative images of ZO-1 expression (green). Nuclei are counterstained with DAPI (blue). (**f**) Densitometric analysis of ZO-1-positive area. Exposure to hyperoxia significantly decreased ZO-1 expression compared to the normoxic controls (**a**,**c**,**f**). The treatment with BRL37344 at 3 mg/kg, but not at 1 mg/kg, had a protective effect (**c**–**f**). No significant changes were observed in the normoxia-exposed pups, treated or not with BRL37344 (**a**,**b**,**f**). Values are expressed as mean ± S.D. *** *p* < 0.001 hyperoxia versus normoxia; °°° *p* < 0.001 hyperoxia + BRL37344 versus normoxia; §§§ *p* < 0.001 hyperoxia + BRL37344 versus hyperoxia. Scale bar = 50 µm.

The lining epithelium of the ileum acts as an essential barrier against external pathogens, and its function depends on the integrity of intercellular junctions [41]. Previous studies have shown that exposure to high oxygen levels in the first few weeks after birth can disrupt the intestinal barrier by affecting these junctions [4]. In the present study, we studied the effect of BRL37344 on tight and adherent junction integrity using immunofluorescent and ultrastructural analysis. As shown in Figure 5, neonatal exposure to hyperoxia reduced the expression of ZO-1, the typical junctional molecule of *Zonulae occludentes* (Figure 5a,c,f). Treatment with BRL37344 at the dose of 3 mg/kg, but not 1 mg/kg, restored ZO-1 expression to the level observed in the normoxic controls (Figure 5a,c–f). Under normoxia, BRL37344 had no effects on ZO-1 expression.

**Figure 6:**
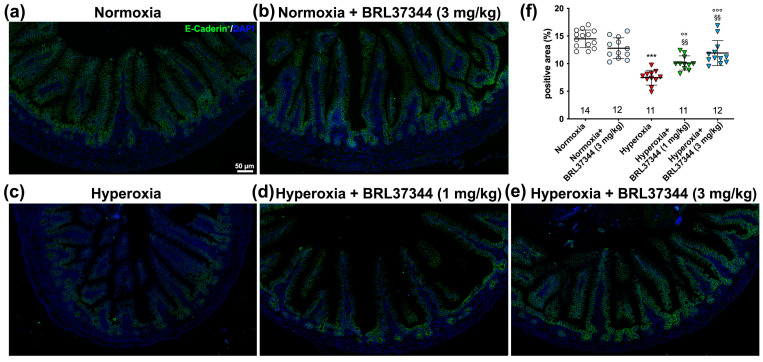
E-cadherin immunolabelling in the ileal mucosa. (**a**–**e**) Representative images of E-cadherin expression (green; nuclei are counterstained blue with DAPI). (**f**) Densitometric analysis of E-cadherin-positive area. Exposure to hyperoxia significantly decreased E-cadherin expression compared to the normoxic controls (**a**,**c**,**f**). The administration of BRL37344 had a partially protective effect (**c**–**f**). No changes can be observed in the normoxic pups treated or not with BRL37344 (**a**,**b**,**f**). Values are expressed as mean ± S.D. *** *p* < 0.001 hyperoxia versus normoxia; °° *p* < 0.01 and °°° *p* < 0.001 hyperoxia + BRL37344 versus normoxia; §§ *p* < 0.01 hyperoxia + BRL37344 versus hyperoxia. Scale bar = 25 µm.

The morphometrical analysis also revealed a statistically significant reduction in the expression of E-cadherin, the typical protein of adherent junctions, in the hyperoxia-exposed controls (Figure 6a,c,f). Treatment with BRL37344 at both 1 mg/kg and 3 mg/kg provided some protection but did not completely prevent this hyperoxia-induced effect. Under normoxia, BRL37344 had no effects on E-cadherin expression (Figure 6a–f).

**Figure 7:**
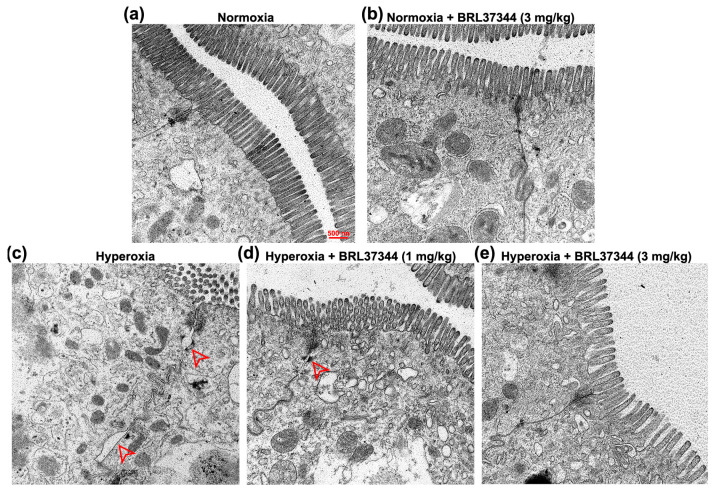
Ultrastructural analysis of intercellular barrier integrity. (**a**,**e**) Representative TEM images of tight and adherent junctions. (**c,d**) Exposure to hyperoxia resulted in increased intercellular spaces of adherent junctions (red arrowhead), an effect prevented by 3 mg/kg BRL37344. (**a**,**b**) No changes were detected in the normoxia-reared rats, treated or not with BRL37344. Scale bar = 500 nm.

The intercellular barrier integrity was then assessed by ultrastructural analysis. The most evident abnormalities were observed in the adherent junctions of the hyperoxia-exposed controls, which displayed a disarrangement of the cytoskeletal–plasma membrane assembly and a marked increase in the intercellular spaces (Figure 7c). Treatment with 3 mg/kg of BRL37344 appeared to preserve the intercellular barrier, while the lower 1 mg/kg dose was ineffective (Figure 7d,e). Once again, no changes were detected in the rats reared under normoxia, treated or not with BRL37344 (Figure 7b).

**Figure 8:**
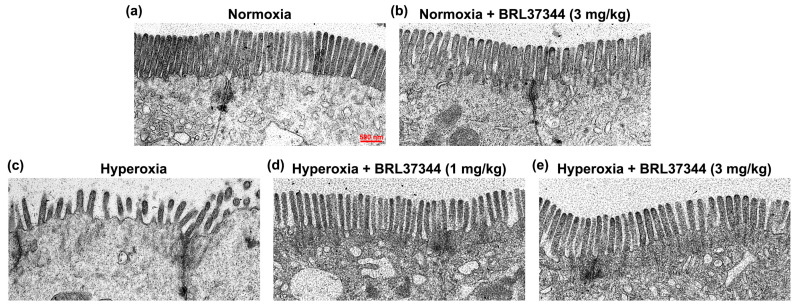
Ultrastructural evaluation of the ileal epithelial brush border. (**a**–**e**) Representative TEM images showing longitudinal sections of microvilli. (**a**,**b**) No changes can be detected in the normoxic pups, treated or not with BRL37344. (**c**,**d**) Hyperoxia exposure resulted in alterations of the microvilli, an effect that was completely prevented by BRL37344 at both doses. Scale bar = 500 nm.

The apical surface of the ileal epithelial cells is covered by microvilli forming the brush border, which plays a role in absorbing nutrients and providing mechanical defense [42]. As shown in Figure 8, exposure to hyperoxia caused a marked disorganization of the brush border, characterized by shorter microvilli and irregular spatial packing (Figure 8a,c). Treatment with BRL37344 at both doses prevented this alteration, preserving the typical morphology of the brush border (Figure 8d,e). Under normoxia, no differences were observed between the pups treated or not with BRL37344 (Figure 8a,b).

### 3.6. Immunofluorescent Analysis of Vascularization

**Figure 9:**
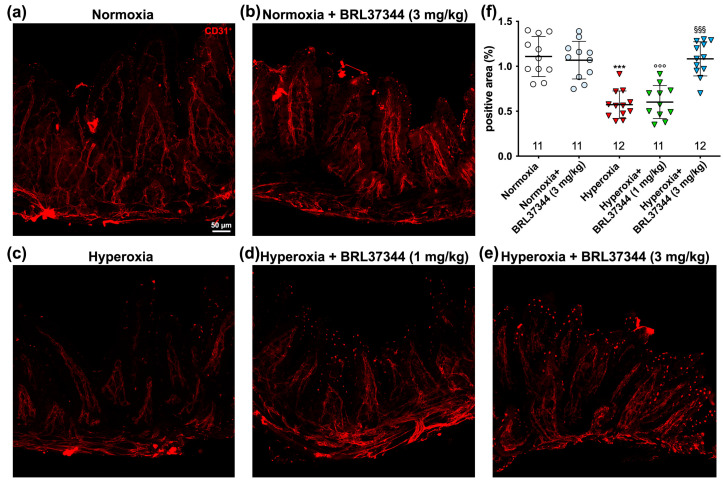
Quantitative analysis of the effects of hyperoxia on vascularization in whole-mount ileal preparations. (**a**–**e**) Representative images of vascular CD31 expression (red). (**f**) Densitometric analysis of CD31^+^ relative area. No differences were found in the normoxic pups, treated or not with BRL37344. The hyperoxia-exposed rats exhibited a significant decrease in CD31^+^ relative area compared to the normoxic controls. BRL37344 at 3 mg/kg prevented this phenomenon. Values are expressed as mean ± S.D. *** *p* < 0.001 hyperoxia versus normoxia; °°° *p* < 0.001 hyperoxia + BRL37344 versus normoxia; §§§ *p* < 0.001 hyperoxia + BRL37344 versus hyperoxia. Scale bar = 50 µm.

Vascularization is crucial for organ development before and after birth and is extensively regulated by oxygen levels [43,44]. Hyperoxia exposure can lead to underdeveloped vascular structures and vaso-obliteration [43,45,46], exposing organs at risk of ischemia and hypoxic stress. To assess the changes in ileal vascularization caused by hyperoxia and the impact of BRL37344 treatment, ileal whole-mount preparations immuno-stained for CD31, a typical vascular marker, were subjected to morphometric analysis. As shown in Figure 9, a significant reduction in the CD31 immunolabelled area, corresponding to blood vessels, was revealed in the ileal submucosa and muscle coat of the hyperoxic controls as compared with the normoxic ones (Figure 9a,c,f). BRL37344 at the 3 mg/kg dose completely prevented this alteration, while 1 mg/kg was ineffective (Figure 9a,c–f). No differences were observed in the normoxic pups, treated or not with BRL37344 (Figure 9a,b,f).

## 4. Discussion

Our previous research demonstrated that exposure to hyperoxia in the first two weeks after birth hinders proximal colon development by decreasing β3-AR expression, while treatment with the β3-AR agonist BRL37344 prevents colonic alterations, likely by preserving the role of this receptor in organ maturation [9]. In the present study, we demonstrate for the first time that the β3-AR agonist BRL37344 exerts a similar protective effect against hyperoxia-induced damage in the ileum.

The first substantial finding of our study is that BRL37344 completely prevented the increase in the plasma levels of 5-*epi*-5-F_2t_-IsoP induced by hyperoxia. This biologically active byproduct of AA peroxidation is considered a reliable biomarker of oxidative stress [47]. Among the different isoprostanes, 5-*epi*-5-F_2t_-IsoP has shown higher concentration in biological fluids and no vasoactive effect compared to the well-known marker of oxidative stress 15-F_2t_-IsoP [48]. It has been previously reported that neonatal exposure to hyperoxia causes oxidative stress, resulting in small bowel damage and epithelial barrier disruption [4]. In this study, we observed that β3-AR activation effectively reduced oxidative stress, as shown by the decreased level of 5-*epi*-5-F_2t_-IsoP with BRL37344 treatment at the 3 mg/kg dose. Our data confirmed the role of β3-AR in reducing oxidative stress, as previously reported in studies on Ewing Sarcoma [49] and chronic heart failure studies [13].

The second relevant finding of this study is that the number of ileal resident β3-AR-positive cells was markedly reduced by hyperoxia while it was preserved by the treatment with BRL37344. This result is consistent with our previous findings in the colon [9] and could improve the understanding of the regulatory mechanism of β3-AR dependent on environmental oxygen levels. The expression of this receptor is known to increase in response to low oxygen levels, both physiologically during fetal development [18,19,50] and in pathological conditions, as occurs in the ischemic proliferative phase of retinopathy of prematurity and in cancer [44]. However, limited information is currently available describing how β3-AR is regulated in response to high oxygen levels. We previously reported that β3-AR expression decreases in the ductus arteriosus (DA) immediately after birth when newborns are exposed to ambient oxygen levels. The increase in oxygen concentration from the prenatal environment to postnatal life is accompanied by a decrease in β3-AR expression, suggesting a potential involvement of this receptor in the closure of the DA [21]. Furthermore, we also demonstrated a reduction in the number of β3-AR-positive cells in the proximal colon of neonatal rats exposed to high oxygen levels [9]. These rats also showed colonic alterations, indicating a possible role of β3-AR in organ development. While not conclusive, these reports, along with the current finding that hyperoxia led to a reduction in the number of ileal β3-AR-expressing cells, suggest that β3-AR can be down-regulated by hyperoxia. This potential down-regulation could have a major impact on the developmental process of some anatomical districts, such as the colon and ileum, in response to varying ambient oxygen levels, potentially making this receptor an innovative pharmacological target for prematurity-related disorders.

In this context, the third major finding of the present study becomes even more important, as it shows the efficacy of BRL37344 (3 mg/kg) in counteracting hyperoxia-induced damage to the ileal mucosa and submucosa. Previous research in the same animal model has revealed that high oxygen levels increase mucosal thickness [7], disrupt the epithelial barrier by affecting the tight junctions, and induce capillary and lacteal dilation [4]. Our present finding further demonstrated that hyperoxia also caused submucosal oedema, leukocyte infiltration, disorganization of the brush border, and loosening of adherent junctions, while treatment with BRL37344 (3 mg/kg) was able to reduce inflammation and preserve the integrity of the lining epithelium. The gut of rodents, which have a short gestation period [51], continues to mature after birth, thus leaving it particularly susceptible to hyperoxia. Excess oxygen levels may impact the development of the ileum by altering the expression of β3-AR, which has an oxygen-dependent regulatory mechanism. Our previous research strongly supports this hypothesis, as it demonstrated the protective effect of β3-AR activation against colonic damage induced by hyperoxia in the same experimental model. Although caution is mandatory when trying to extrapolate the results from animal studies to humans, our finding that a β3-AR agonist preserved the integrity of the gut surface epithelium could have significant clinical implications, particularly in the prevention of necrotizing enterocolitis (NEC), in which an impaired intestinal barrier predisposes patients to bacterial leakage [52]. Of note, despite its beneficial effect in preserving the integrity of the ileal lining epithelium, BRL37344 did not prevent the body weight loss induced by hyperoxia. As previously explained [9], this outcome is likely due to the general harmful effects of oxidative stress and the correlated inflammation rather than the recognized anorexic effect of β3-AR activation [53,54]. This hypothesis is further supported by the finding that administering BRL37344 under normal oxygen conditions did not affect the body weight of the pups.

An unexpected result of this study is that hyperoxia led to mucin over-production by goblet cells, as indicated by the increased PAS^+^ relative area within the lining epithelium. While specific data on ileal mucin production in the hyperoxic model we used are currently unavailable, other animal models related to prematurity, such as fetal exposure to maternal inflammation (FEMI) and NEC, have reported a decrease in the number of goblet cells [55,56,57]. This apparent discrepancy between our and previous findings on mucin production and goblet cell number can be attributed to the fact that we evaluated the relative area of PAS-positive staining within the epithelium without directly counting the number of goblet cells. Of note, these two parameters are not necessarily correlated. Additionally, the difference could also be due to the distinct pathogenic mechanisms involved. FEMI and NEC models involve a direct inflammatory response, mainly through the lipopolysaccharide receptor Toll-like receptor 4, resulting in intestinal barrier breakdown and subsequent bacterial translocation [58]. Conversely, hyperoxia primarily induces oxidative stress and dysbiosis, which in turn activate intestinal inflammation in a vicious cycle leading to increased ROS production and, ultimately, mucosal damage [59,60]. Moreover, the remodeling of the microbiota in favor of the increase in aerobic species prompts the alteration of the barrier function and subsequently causes infections by commensal and pathogenic species. In this scenario, the over-production of mucin may represent an acute defense mechanism against hyperoxia-induced mucosal damage. If exposure to high oxygen levels persists, it may lead to impairment and loss of goblet cells and a decrease in mucin production, potentially contributing to NEC development. It is noteworthy that supplemental oxygen therapy for preterm neonates with respiratory syndrome is associated with an increased risk of developing NEC [61].

A final, remarkable result of the present study is that hyperoxia deeply impaired the ileal vascularization, while the treatment with BRL37344 at 3 mg/kg effectively prevented this alteration. During the early postnatal period, the gut undergoes substantial remodeling, resulting in major changes in the overall structure and vascular network of the villi [62]. While vasculogenesis mainly occurs during early embryonic development [63], fetal and postnatal vascularization is primarily due to angiogenesis and results in a complex network of microvessels that extends from the lamina propria into the center of the villus up to the tip and forms an interconnected system of capillaries. Enclosed within this “capillary cage”, a blunt-ended lacteal emerges during embryogenesis and continues to develop after birth [64]. Our study first shows that exposure to neonatal hyperoxia significantly reduced the vascularization of the ileum and resulted in lacteal dilation, and that both phenomena were completely prevented by the administration of BRL37344 at a dosage of 3 mg/kg. While it has been documented that hyperoxia can induce lacteal dilation [4], its impact on vascularization is debated. A single report suggests that hyperoxia can induce angiogenesis in a few cases [12]. In contrast, many studies agree that hyperoxia causes death and reduced proliferation of vascular cells, such as human microvascular endothelial cells, ultimately leading to microvascular dysfunction [46,65,66,67]. Moreover, studies carried out on oxygen-induced retinopathy (OIR), a model for human retinopathy of prematurity (ROP), have provided further evidence of the negative effects of hyperoxia on vascularization during postnatal organ development. These studies have shown that the increased oxygen levels during the early hyperoxic phase of OIR down-regulated the production of proangiogenic factors, thus impairing vascularization [6]. In contrast, during the second phase of ROP, known as hypoxic or proliferative, low oxygen promotes the upregulation of proangiogenic factors and the induction of aberrant neovascularization of the retina [68]. Even in the retina, sudden exposure to a relatively hyperoxic environment, such as after birth, leads to a rapid down-regulation of the expression of β3-ARs [22], indicating that the environmental oxygen level may regulate vascularization by affecting β3-AR. This hypothesis is also based on the understanding that hypoxia-inducible factor (HIF)-1, the primary transcriptional activator dependent on oxygen levels, has been found to enhance β3-AR expression by directly binding to its enhancer region [50]. Taken together, these findings indicate a potential connection between high environmental oxygen levels, down-regulation of β3-AR expression, and decreased vascularization. The intriguing interplay between these factors may have significant clinical implications for prematurity-related conditions. Our findings demonstrate that treatment with BRL37344, a β3-AR agonist, could uncouple the impact of the environmental oxygen levels from vascularization, thus effectively protecting the ileal blood vessels from the effect of hyperoxia. Therefore, it is reasonable that the pharmacological targeting of β3-AR could be effective not only in preventing NEC but also in treating other conditions specific to preterm infants, such as BPD and periventricular leukomalacia, where hyperoxia promotes abnormal vascular remodeling and consequent hypoplasia of the lung [69,70] and central nervous system [71] parenchymas, respectively. Our results suggest that treatment with a β3-AR agonist could be a valuable pharmacological strategy for preventing prematurity-related diseases. Of note, further research is needed to validate this hypothesis.

Exposure to a hyperoxic environment is known to promote the development of gut dysbiosis [72]. This effect persists in mice through adolescence [73] and is commonly observed in human preterm infants [74]. Maintaining intestinal vascularization through pharmacological β3-AR agonism may likely have a beneficial effect on the composition of the intestinal microbiome in preterm neonates.

A final point deserving comment is the marked mortality of the pups caused by the treatment with BRL37344 at the highest dose (6 mg/kg). As previously reported, one potential explanation is that BRL37344, administered at 6 mg/kg, is toxic [9]. Another potential reason is that the combination of hyperoxia-induced weight loss, abnormal feeding behavior due to the anorexic effect of β3-AR, and sub-lethal toxic effects of 6 mg/kg of BRL37344 produced a synergistic effect lethal to the pups. For ethical reasons, we opted not to test this high dose of BRL37344 in normoxic pups. When BRL37344 was administered at the effective dose of 3 mg/kg to prevent hyperoxia-induced alterations, it did not exhibit anorexic or toxic effects or modify any of the analyzed parameters. These findings indicate that BRL37344 has a therapeutic window at low doses.

This study has some limitations. Our choice to use BRL37344 in the experiment was based on its 20-fold greater selectivity for β3-AR over β2-AR in humans [75] and its higher affinity for the rodent β3-AR receptor [76]. Furthermore, its compatibility with subcutaneous administration [77] aligns with the requirements of the animal model, which requires neonatal rats and does not allow for intravenous or oral treatments. However, more recently, it has been demonstrated that BRL37344 also acts via β2-AR in restricted tissues, such as skeletal muscle [78]. Consequently, we cannot rule out that some of the effects documented in this study could also be mediated via β2-AR. To overcome this issue, additional experimental studies should be carried out to evaluate the outcomes of more selective, second-generation agonists such as mirabegron or by using β3-AR KO animals. A second relevant limitation of this study is that the effect of hyperoxia on changing the composition of the gut microbiome has not been taken into account. The microbiota is a key factor that may influence ileal maturation processes. However, the research on dysbiosis is limited by the high heterogeneity of the data from the literature [72] and, anyway, requires different in vivo experimental approaches. The last significant limitation of the present study is the absence of a functional assessment of the mucosal integrity. This analysis could provide a better understanding of the positive impact of BRL37344 in counteracting the hyperoxia-induced ileal damage. Further morpho-functional evaluations are needed to comprehensively assess the complex relationship between ileal mucosal integrity, β3-AR expression, and the gut microbiota.

Findings from animal studies require extreme caution when extrapolated to humans, particularly when involving extreme conditions such as high oxygen stress. However, the results indicating that β3-AR agonists protect against hyperoxia-induced ileal damage could have promising implications in clinical practice for counteracting the side effects of supplemental oxygen therapy. Additionally, given the known connection between hyperoxia and intestinal damage [79,80], which increases the risk of NEC [41], our data suggest that pharmacological targeting of β3-AR could also be a promising approach for the prevention and treatment of this disease.

## 5. Conclusions

In conclusion, the current study shows that neonatal exposure to high oxygen levels in rat pups significantly damages the ileum, possibly by decreasing β3-AR expression. Furthermore, our findings provide the first evidence that, if administered at an appropriate dose, the selective β3-AR agonist BRL37344 can exert effective protection. Overall, these findings further support the potential use of drugs targeting β3-AR as a new, valuable pharmacological approach for treating preterm infants against the common adverse effects of supplemental oxygen therapy, such as bronchopulmonary dysplasia, necrotizing enterocolitis, retinopathy of prematurity, and periventricular leukomalacia.

## Figures and Tables

**Table 1:** The body weight of pups at birth (P0) and the end of the experimental period (P14).

Experimental Group	Age (d)	Body Weight (g)	Age (d)	Body Weight (g)
Normoxia	0	6.4 ± 0.1	14	33.1 ± 0.6
Normoxia + BRL37344 (3 mg/kg)	0	6.5 ± 0.2	14	31.9 ± 1.1
Hyperoxia	0	6.3 ± 0.2	14	21.0 ± 0.8 ***
Hyperoxia + BRL37344 (1 mg/kg)	0	6.3 ± 0.3	14	21.7 ± 0.7 °°°
Hyperoxia + BRL37344 (3 mg/kg)	0	6.4 ± 0.2	14	23.6 ± 1.6 °°°

Values are expressed as mean ± S.D. *** *p* < 0.001 hyperoxia versus normoxia; °°° *p* < 0.001 hyperoxia + BRL37344 versus normoxia.

## Data Availability

The datasets generated during and/or analyzed during the current study are available from the corresponding author upon reasonable request.
